# Blown away? Wind speed and foraging success in an acoustic predator

**DOI:** 10.1007/s13364-023-00673-7

**Published:** 2023-02-13

**Authors:** Samantha Renda, Stéphanie Périquet, Aliza le Roux

**Affiliations:** 1 Department of Zoology and Entomology, University of the Free State, Qwaqwa campus, Private Bag X13, Phuthaditjhaba, Free State Province 9866 South Africa; 2 Ongava Research Centre, Private Bag 12041, Suite No. 10 Ausspannplatz, Windhoek, Namibia

**Keywords:** Acoustic, Bat-eared fox, Foraging, *Hodotermes mossambicus*, *Otocyon megalotis*, Prey detection, Sensory ecology

## Abstract

Foraging animals must contend with fluctuating environmental variables that affect foraging success, including conditions like wind noise, which could diminish the usefulness of particular sensory modes. Although the documented impact of anthropogenic noise on animal behavior has become clear, there is limited research on natural noise and its potential influence on mammalian behavior. We investigated foraging behavior in the myrmecophagous bat-eared fox (*Otocyon megalotis*), a species known to rely predominantly on hearing for prey detection. For a year, we monitored the foraging behavior of 18 bat-eared foxes from a habituated population in South Africa, amidst varying wind speeds (0–15.5 km/h). In contrast to expectations, foraging rates did not generally decline with increasing wind speed, except for foraging rate outside termite patches in fall. Furthermore, wind speed had little correlation with time spent in patches. In winter, however, we observed an increase in foraging rate with increasing wind speed both within and outside patches. At the observed wind speeds, these acoustically driven insectivores continue to forage effectively despite potentially distracting or masking noises. With anthropogenic noise producing sound across a broader frequency range, it is important to examine the responses of these canids to artificial sources of acoustic disturbance as well.

**Supplementary Information:**

The online version contains supplementary material available at 10.1007/s13364-023-00673-7.

## Introduction

Predators rely on various sensory cues from their prey to successfully detect food items. The salience of different cues varies both with the ecology of the predator (Klinka and Reimchen 2009), and with ambient environmental conditions. Changes in factors such as light intensity, vegetation density, or noise levels affect the utility of sensory cues (Barber et al. 2010; Goerlitz et al. 2008; Klinka and Reimchen 2009). In visual hunters such as pikes (*Esox lucius*), for example, increasing water turbidity results in reduced reaction distance when attacking prey (Ranåker et al. 2012), while black-backed jackals (*Canis mesomelas*) increase hunting behavior and exploration of risky anthropogenic habitats during brighter, full moon nights (Botha et al. 2022).

Most of the current research on acoustic disturbance has focused on the effect of anthropogenic noise (Shannon et al. 2016), particularly as it affects communication, mating, or vigilance patterns (Barber et al. 2010; Quinn et al. 2006; Reijnen et al. 1997). Much less is known about the impact of natural sounds—geophonies like water and wind—on foraging behavior in acoustic hunters (Gomes et al. 2021). For many nocturnal insectivores, acoustic cues are of paramount importance in prey detection (*Microcebus murinus*, Goerlitz and Siemers 2007; *Myotis myotis*, Schaub et al. 2008, *Otocyon megalotis*, Renda and le Roux 2017). The auditory sense enables such hunters to locate prey not only from a distance but also in visually cluttered environments (Goerlitz and Siemers 2007). Visually inconspicuous prey may therefore still be detected through pin-pointing their location aurally, often with the aid of over-sized pinnae (Ramsier and Dominy 2012). Foragers reliant on this sense are also subject to ambient interferences: noise, both of anthropogenic and natural origin, plays a role in obstructing prey-generated sounds (Schaub et al. 2008) and has been shown to reduce the prey-detection efficiency of predators such as greater mouse-eared bats (*Myotis myotis*) and Daubenton’s bats (*Myotis daubentonii*) (Siemers and Schaub 2011; Luo, Siemers, and Koselj 2015). Schaub et al. (2008) revealed that both anthropogenic and natural ambient noise resulted in lower foraging success in greater mouse-eared bats. Interestingly, a greater deleterious effect was noted from simulated wind-generated noise than from anthropogenic noise. The disruptions brought on by noise are not necessarily due to acoustic masking (overlapping with sensory cues used to detect prey), but can sometimes be ascribed to acoustic distraction, which interferes with hunting success (Allen et al. 2021). Natural environmental noise may disrupt foraging behavior even in non-specialist foragers like California ground squirrels*, Otospermophilus beecheyi* (Le et al. 2019). Given the potential of geophonies to shape behavior, this is a rich area for further behavioral research and one that has received little attention in comparison to anthropogenic noise.

Bat-eared foxes (*Otocyon megalotis*, “foxes” hereafter) are nocturnal insectivores that feed primarily on termites, predominantly harvester termites, *Hodotermes mossambicus*, although a variety of other prey items are consumed as and when they become available (Jumbam et al. 2019; Kuntzsch and Nel 1992; Malcolm 1986). Whereas foxes’ habitat selection patterns do not closely mirror those of the harvester termites (Périquet and le Roux 2018), their primary prey items are invertebrates that do not produce conspicuous warning or advertising sounds (Grant and Samways 2015). These canids have been observed to use predominantly auditory stimuli when seeking prey under natural (Grant and Samways 2015; Malcolm 1986) and experimental conditions (Renda and le Roux 2017). The disproportionately small olfactory turbinals in fox skulls further underscore that they rely less on olfactory cues compared to other terrestrial canids (Green et al. 2012). Given their reliance on audition in finding acoustically inconspicuous prey (Goerlitz et al. 2008), we would therefore expect wind to have a detrimental effect on their foraging success. In this study, we recorded foraging behavior of known individual foxes along with wind speed used as a proxy for ambient noise levels (cf. Hayes and Huntly 2005). We anticipated that higher wind speeds, inducing higher levels of ambient noise, would hinder foxes’ prey detection and more specifically that:

- The foraging rate (number of successful foraging events per unit of time) outside termite patches should decrease with increasing wind, aligned with an increase in ambient noise.
- The foraging rate within termite patches should decrease with wind as foxes would continue feeding in patches for longer, as these are predictable sources of food, once the patch is discovered.
- Foxes should spend more time feeding in termite patches under windy conditions due to the difficulty of detecting single prey items outside these relatively rich areas, and the effect should be more prominent in winter when overall arthropod availability drops.

## Materials and methods

### Study site and population

We observed habituated foxes from a wild population in the Kuruman River Reserve (28°580 S, 21°490 E), Northern Cape province, South Africa. Vegetation in the reserve consists of scattered camel thorn trees (*Acacia erioloba*) grading out into dry scrubland and sparsely vegetated dunes. The reserve is characterized by four seasons, based on temperature and rainfall. Winters (June to August) are usually dry and cold, with temperatures often below 0°C during the night, while summers (December to February), when most of the precipitation occurs (~250mm/year), can be extremely hot (40°C during daytime). Foxes were fully habituated to the presence of observers following them on foot at night (foxes became very wary of human observers during the day) from a few meters away (Renda and le Roux 2017). Observers could identify foxes individually thanks to natural markings, and VHF radio-collars on a few individuals (*n*=8). We followed known individuals only once a week, for a 2-h session between dusk and dawn, to reduce disturbance. In these “follow” sessions, we noted a wide range of prey items being eaten, including termites (Isoptera: Hodotermitidae), ants (Hymenoptera: Formicidae), beetles (Coleoptera: Carabidae; Tenebrionidae; Scarabaeidae), antlions (Neuroptera: Myrmeleontidae), Lepidoptera (various families), Arachnida (various families), and some mammalian vertebrates (Rodentia: Muridae), as well as occasional amphibians or reptiles. Despite their relatively flexible diets, this study population was confirmed termite specialists (Jumbam et al. 2019).

### Behavioral observations and foraging parameters

We conducted our observations of 18 adult foxes (8 females and 10 males) between July 2014 and April 2016. While observer presence could have had a potential effect on prey behavior (e.g., they might fly away or cease movement), this effect is unlikely to cause any patterns in our results since it would have been present during all our observations. The same remark is true concerning the potential effect of observer on fox behavior and noise resulting from observer movement (though noise from observers was minimized insofar as possible). Ten minutes after finding a fox, we would start data recording, using the program Cybertracker (www.cybertracker.org) loaded on an Android tablet (Samsung Galaxy). We noted the date, time, and GPS coordinates of each successful foraging instance, including a description of the item(s) eaten, when clearly visible to the observer.

When foxes foraged on termites, it was not possible to count the exact number of termites eaten. In this case, we characterized successful foraging events using “productive steps.” We defined a “productive step” as a step (displacement of either front limb) during which at least one termite was eaten. We further defined termite “foraging bouts” as at least 15 consecutive productive steps (typically with multiple termites eaten per step), grouping bouts within 20 meters and 5 minutes of one another into larger, contiguous “patches.” This approach allowed us to conservatively quantify termite-feeding bouts, which were periods of focused movement within a relatively small space as termites close to a nest entrance were snapped up. These bouts were different from pouncing, scratching, or coursing movement when foxes hunted other prey or moved between patches. For analysis, we treated individual termite patches as discrete foraging events, as we presumed foxes to be less reliant on audition when foraging on termites in this manner—termites were typically active in dense concentrations on the surface. Across all observation sessions (hereafter referred to as “follows”), we identified 413 termite patches. We computed foraging rate inside each termite patch using the number of productive steps within a given patch divided by the time spent in this patch, and calculated total time spent within termite patches for each follow.

Whereas we measured foraging rate for every single patch used, foraging rate outside patches was calculated per hour of observation time. Outside termite patches, each foraging event corresponded to the consumption of a discrete prey item and we thus calculated the daily foraging rate outside termite patches as the total number of foraging events (i.e. number of items eaten) outside patches, divided by total time spent outside patches. For this measure, we pooled all prey items together. The datasets analyzed during this study are available from the corresponding author on reasonable request.

### Meteorological data

We collected meteorological data from an on-site weather station at the Kuruman River Reserve field station, roughly at the center of our study area. The station recorded hourly wind speeds at a height of 2 m above-ground, and temperature throughout the year. The maximum recorded wind speeds were 15.5 km/h (classified as a Beaufort value of 3 or “gentle breeze”) for all observation sessions. According to the Beaufort scale, wind speeds of 6 km/h upwards would start generating noise through light leaf rustling, up to more pronounced noise produced by moving branches and grasses (World Meteorological Organization 2016). Average wind speed across all behavioral observations was 3.8 km/h (SD= 3.8). Considering the relative homogeneity of the landscape at the study site, we assumed that wind speed would be an effective proxy for noise level, similar to the strong correlation between wind speed and noise spectrum (from 500 Hz-8,000Hz) in shallow water (Murugan, Natarajan, and Kumar 2011). As temperature is a factor known to affect arthropod activity (Heatwole 2012), and we did not want to add temperature as a possible confounding variable, we cut all data corresponding to temperatures below 10 °C from the dataset, based on the thermal limits of foraging *Hodotermes* workers (Mitchell et al. 1993).

### Statistical analyses

We performed all data analyses in the R statistical environment (version 3.5.1, R Core Team 2016). We assessed the effect of the interaction between wind and season on foraging rate outside and inside termite patches using Linear Mixed Effects Models (LMMs), with each follow’s identity (i.e., date and time stamp) nested in individual as a random intercept. To meet model assumptions, we log-transformed foraging rates outside and inside patches. We ran LMMs using the “nlme” package (version 3.1-137, Pinheiro et al., 2007).

We used Generalized Linear Mixed Effects Models (GLMMs) to study the effect of the interaction between wind and season on the time spent in patches (per hour of observation/follow), using follow duration (log-transformed) as an offset and follow identity nested within individual as a random intercept. As models using a Poisson error distribution showed high overdispersion, we used a negative binomial error distribution for the final models. We fit GLMMs with the package “glmmADMB” (Fournier et al. 2012; Skaug et al. 2016). Following Zuur and Ieno (2016), we verified model assumptions by plotting residuals versus fitted values, versus each covariate in the model and versus each covariate not in the model. We compared models to a null model using likelihood ratio tests (LRTs) to assess model fit.

## Results

### Foraging rate outside termite patches

**Fig. 1:**
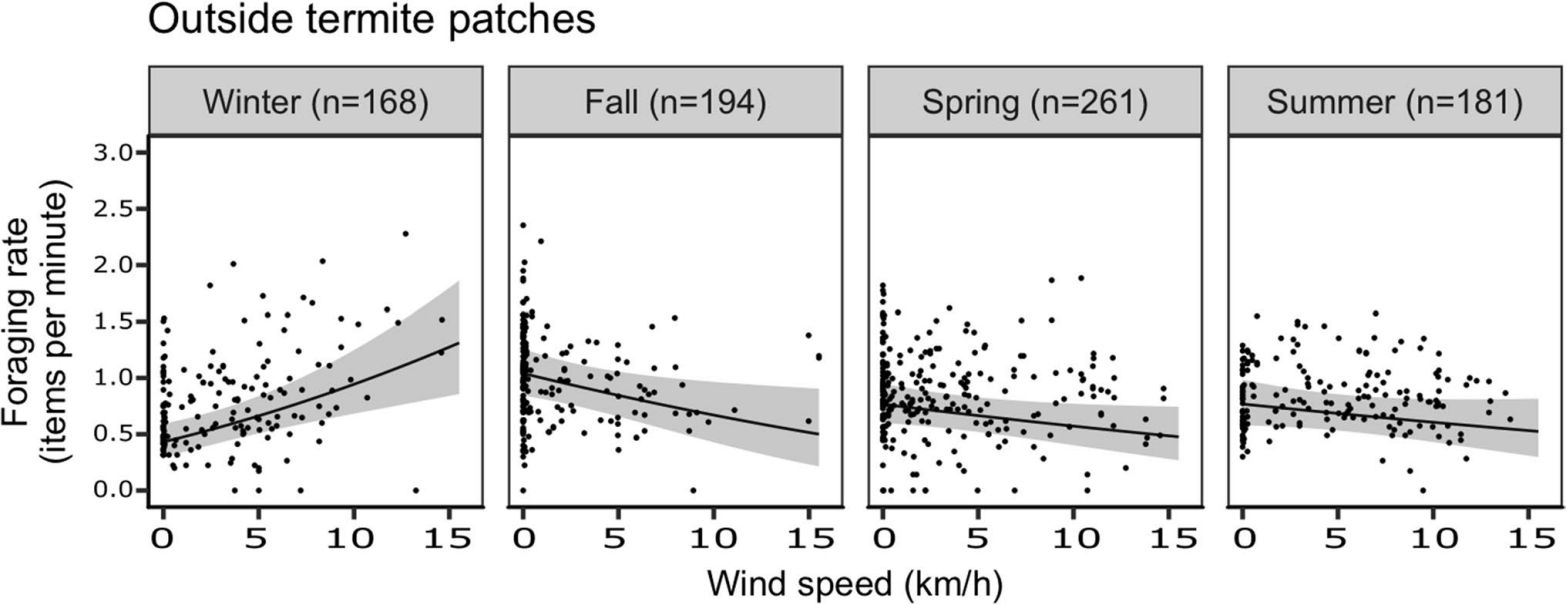
Correlations between wind and foraging rate outside termite patches of bat-eared foxes during the four seasons. Numbers in parenthesis indicate sample size (focal hours) for each season. Values are predicted from the GLMM with 95 % confidence intervals (grey bands). Dots show the actual data.

We extracted foraging rates outside termite patches for a total of 804 focal hours. A model including the wind-season interaction performed better than the null model (LRT: *χ*^2^= 39.1, *P* <0.001). The interaction wind-season was significant (*χ*^*2*^ = 23.4, *P* <0.001) with wind having a significant positive effect on foraging rate in winter (*P* <0.001, Table 1) and negative in all other seasons (Table 1, Fig. 1).

**Table 1:** Model parameter estimates for bat-eared foxes’ foraging rate outside and inside termite patches, and the time spent inside these patches

	Foraging rate outside patches				Foraging rate inside patches				Time inside patches			
Parameter	Estimate	SE	*t*-value	*P*-value	Estimate	SE	*t*-value	*P*-value	Estimate	SE	*z*-value	*P*-value
Intercept (Winter)	0.66	0.06	11.84	<0.001	1.94	0.14	14.15	<0.001	−1.306	0.274	−4.770	<0.001
Fall	0.35	0.06	5.69	<0.001	0.32	0.14	2.27	0.025	0.211	0.274	0.771	0.441
Spring	0.22	0.05	4.09	<0.001	0.35	0.15	2.37	0.020	−0.087	0.290	−0.299	0.765
Summer	0.22	0.06	3.53	<0.001	0.21	0.17	1.28	0.204	0.140	0.327	0.429	0.668
Wind	0.03	0.01	3.96	<0.001	0.02	0.02	1.16	0.248	0.053	0.040	1.327	0.185
Fall × wind	−0.05	0.01	−4.35	<0.001	−0.03	0.02	−1.57	0.118	−0.049	0.045	−1.074	0.283
Spring×wind	−0.05	0.01	−4.63	<0.001	−0.01	0.02	−0.58	0.559	−0.046	0.045	−1.020	0.308
Summer×wind	−0.04	0.01	−3.93	<0.001	−0.03	0.02	−1.45	0.148	−0.069	0.047	−1.469	0.142

### Foraging rate inside termite patches

**Fig. 2:**
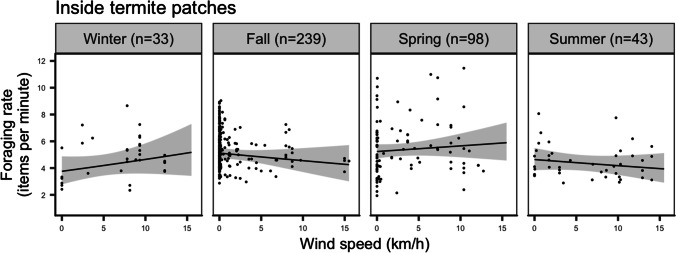
Links between wind and foraging rate inside termite patches of bat-eared foxes during the four seasons. Numbers in parenthesis indicate sample size (focal hours) for each season. Values are predicted from the GLMM with 95 % confidence intervals (grey bands). Dots show the actual data.

We extracted foraging rates inside termite patches for a total of 813 focal hours. The model including the wind-season interaction was significantly better than the null one (LRT: *χ*^*2*^ = 14.7, *P* =0.04). Only season had a significant effect (*χ*^*2*^ = 9.6, *P* = 0.02, Fig. 2) on foraging rate. Foraging rate in termite patches in fall and spring was significantly higher than in winter (*P* = 0.03, Table 1, Fig. 2).

### Time spent in termite patches

We recorded a total of 204 bouts of foraging in termite patches. The model including the wind-season interaction was not significantly better than the null model (LRT: *χ*^*2*^ = 7.0, *P* = 0.43), suggesting that wind had no effect on the time foxes spent foraging on termites in patches.

## Discussion

We used wind speed as a proxy for ambient noise and expected that stronger winds would negatively affect bat-eared foxes’ foraging behavior due to their reliance on sound to detect prey. Contrary to our predictions, wind did not have a deleterious effect on foraging rates, and in fact correlated with increased foraging rates in winter. Although wind did have a significant negative effect on foraging outside of termite patches in all seasons compare to winter, this effect was very small (see Fig. 1) and barely negative, and thus may not be biologically relevant in terms of the actual intake of a foraging fox. Interestingly, in winter, foraging rate increased significantly with wind speed, both within and outside patches. This effect may have been mediated by a shift in fox ecology peculiar to the winter season, as foxes’ activity patterns closely mirror that of their termite prey, similar to other myrmecophagous mammals (e.g., Abba and Cassini 2010). In southern Africa, foxes often exhibit a more diurnal foraging pattern in winter (Lourens and Nel 1990; Nel 1990), linked to a diurnal shift in harvester termite activities (Nel 1990). Additionally, desert day-time wind speeds are typically higher in winter, and foxes have been noted to decrease diurnal foraging as winds increase, with activity often ceasing altogether at high speeds (Lourens and Nel 1990). Windy days during the current study followed this trend, with winter wind speeds typically higher earlier in the day and tapering off by evening (see Supplementary Information). It is likely, therefore, that foxes in the present study missed diurnal foraging opportunities more frequently during windy winter days. With winter being a relatively food-constrained season (Jumbam et al. 2019), foxes presumably experienced much higher motivation to forage more effectively during nights following windy days.

In our third prediction, foxes were expected to spend more time in termite patches under windy conditions as wind noise would increase the difficulty of finding alternate patches or food items. Again, the amount of time foxes spent in termite patches did not increase, and there appeared to be no greater value to remaining in patches as wind speeds rose. Although information is limited, sensory information is known to influence patch use in both invertebrates and vertebrates. For wolf spiders (*Schizocosa ocreata*), patches containing clear sensory signals indicating the presence of prey elicited longer residence times than control patches (Persons and Uetz 1996). Similarly, common brushtail possums (*Trichosurus vulpecula*) spent more time foraging in patches where olfactory cues indicate richer food sources being available (Mella et al. 2018). However, up to the moderate wind speeds recorded in this study, foxes did not alter their patch residence times. This may align with Mankin and Benshemesh’s (2006) findings that subterranean acoustic cues from disturbed ant and termite nests remain detectible (by geophone) at close range even amidst wind gusts. As foxes are sensitive to ambient light conditions (Welch et al. 2017), and substrate-borne vibrational cues may also be detectible through paws rather than pinnae (Mason and Wenger 2019), we also cannot eliminate the possibility that foxes increase reliance on other sensory modalities during windy nights.

It could be argued that wind alters prey behavior and that the concomitant decline in patch profitability would result in foxes abandoning patches earlier despite out-of-patch sensory constraints. However, though the impact of wind on *Hodotermes* worker activity specifically has not been quantified, studies on harvester termites with similar foraging ecology have revealed little effect of wind on activity levels. Workers of *Trinervitermes*, for example, were found to continue foraging at wind speeds in excess of 21.6 km/h (Adam et al. 2008). Similarly, wind was found to have little effect on the foraging behavior of *Baucaliotermes* workers (Geerts et al. 2016). Comparable results have been found for other common arthropod prey items of foxes including ants (Hymenoptera:Formicidae) and beetles (Coleoptera:Tenebrionidae, Briese and Macauley 1980; Curtis, 1985; Heatwole 2012). Wind may even have a stimulatory effect on some scorpions (Scorpiones: Buthidae), beetles (Coleoptera: Tenebrionidae), and antlions (Neuroptera: Myrmeleontidae), particularly at lower speeds i.e., < 9 km/h for antlions (Heatwole 2012; Skutelsky 1996; Szentkirályi et al. 2005). Where inhibition of arthropod activity does occur, this is often at higher levels (upwards of 14.4 km/h for ants; Heatwole 2012), very close to the maximum of 15.5 km/h recorded in the current study. We are aware that the range of wind speeds under consideration here is not very wide, but as foraging observations were conducted on a daily basis throughout the year, we believe these wind speeds are representative of the overall conditions in which foxes forage in the study area.

It may appear counterintuitive that the naturally occurring geophony of wind did not affect the foraging behavior of an acoustic hunter like the bat-eared fox. This contrasts with the known and significant impacts of white noise and anthropogenic sounds (technophonies) on other mammals’ foraging behavior (Gomes et al.,^2021^; Shannon et al. 2016). It may be that we did not measure the biologically salient responses of foxes to wind noise. Similar to foraging bats (Schaub et al. 2008), foxes may have moved to microhabitats with less pronounced acoustic disturbance. We did not measure actual noise levels in different habitat and wind conditions so we cannot discuss this possibility. Foxes may also simply stop foraging once noise levels increase beyond a certain threshold: on a few occasions of very strong wind, we noticed that foxes stopped foraging and rested, or took flight in the presence of observers, suggesting some compromise of their auditory awareness. A final consideration, however, is that bat-eared foxes are well-adapted to the natural disturbance of even moderate winds. As anthropogenic noise often disrupts natural soundscapes across a range of frequencies that differs from environmental and biological sources of sound (Farina 2019; Gomes et al. 2021), this acoustic adaptation of bat-eared foxes—and those of other acoustic hunters—may be severely tested with continuing global change.

## Supplementary information

ESM 1
