# Supplementary material for: Blown away? Wind speed and foraging success in an acoustic predator
Source: Mamm Res. 2023 Feb 13;68(2):215–21. doi: 10.1007/s13364-023-00673-7 (PMC10033565; doi:10.1007/s13364-023-00673-7)
Supplement: Supplementary file 1 — ESM 1 [file 13364_2023_673_MOESM1_ESM.docx]

**SUPPLEMENTARY INFORMATION**


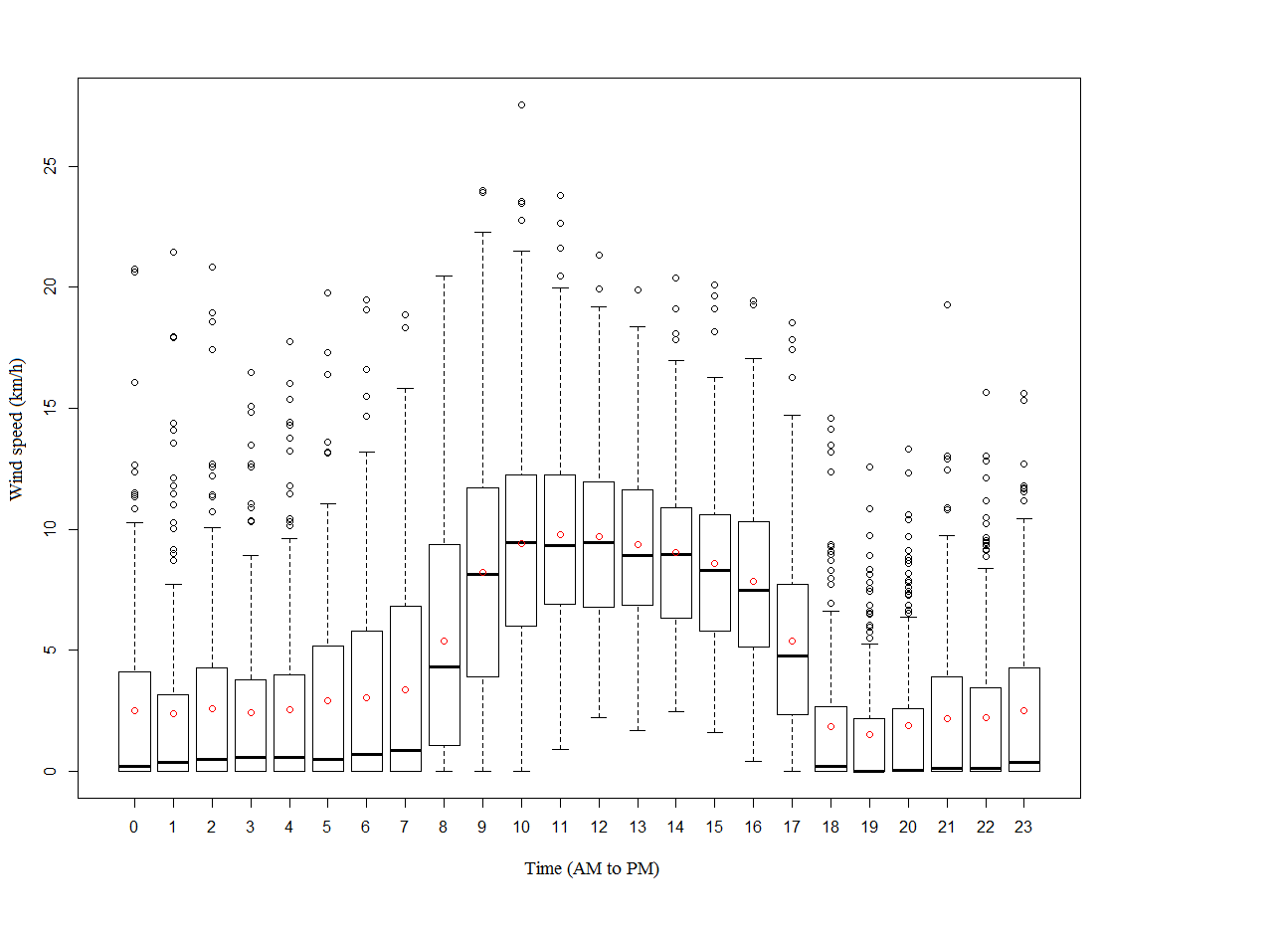


**Figure 3.** Winter wind speeds by hour of the day at the Kuruman River Reserve, in the Kahalari Desert, South Africa. Red points indicate mean wind speed for a particular hour for the entire winter season.
